# Next Generation Sequencing for the Analysis of Parvovirus B19 Genomic Diversity

**DOI:** 10.3390/v15010217

**Published:** 2023-01-12

**Authors:** Federica Bichicchi, Niccolò Guglietta, Arthur Daniel Rocha Alves, Erika Fasano, Elisabetta Manaresi, Gloria Bua, Giorgio Gallinella

**Affiliations:** 1 Department of Pharmacy and Biotechnology, University of Bologna, 40138 Bologna, Italy; 2 Laboratory of Technological Development in Virology, Oswaldo Cruz Foundation/FIOCRUZ, Brasil Avenue 4365, Manguinhos, Rio de Janeiro 21040-900, Brazil; 3 Microbiology Section, IRCCS Sant’Orsola Hospital, 40138 Bologna, Italy

**Keywords:** Parvovirus B19, genetic diversity, viral quasispecies, Next Generation Sequencing, Shannon Entropy, cluster analysis

## Abstract

Parvovirus B19 (B19V) is a ssDNA human virus, responsible for an ample range of clinical manifestations. Sequencing of B19V DNA from clinical samples is frequently reported in the literature to assign genotype (genotypes 1–3) and for finer molecular epidemiological tracing. The increasing availability of Next Generation Sequencing (NGS) with its depth of coverage potentially yields information on intrinsic sequence heterogeneity; however, integration of this information in analysis of sequence variation is not routinely obtained. The present work investigated genomic sequence heterogeneity within and between B19V isolates by application of NGS techniques, and by the development of a novel dedicated bioinformatic tool and analysis pipeline, yielding information on two newly defined parameters. The first, α-diversity, is a measure of the amount and distribution of position-specific, normalised Shannon Entropy, as a measure of intra-sample sequence heterogeneity. The second, σ-diversity, is a measure of the amount of inter-sample sequence heterogeneity, also incorporating information on α-diversity. Based on these indexes, further cluster analysis can be performed. A set of 24 high-titre viraemic samples was investigated. Of these, 23 samples were genotype 1 and one sample was genotype 2. Genotype 1 isolates showed low α-diversity values, with only a few samples showing distinct position-specific polymorphisms; a few genetically related clusters emerged when analysing inter-sample distances, correlated to the year of isolation; the single genotype 2 isolate showed the highest α-diversity, even if not presenting polymorphisms, and was an evident outlier when analysing inter-sample distance. In conclusion, NGS analysis and the bioinformatic tool and pipeline developed and used in the present work can be considered effective tools for investigating sequence diversity, an observable parameter that can be incorporated into the quasispecies theory framework to yield a better insight into viral evolution dynamics.

## 1. Introduction

Within the family *Parvoviridae*, Parvovirus B19 (B19V) is a widespread human virus, responsible for an ample range of clinical manifestations [1]. B19V is mostly transmitted through the respiratory route, while the major tropism is towards erythroid progenitor cells in bone marrow (EPCs) that are susceptible and permissive to viral replication, dependent on their differentiation state and replicative rate [2]. Infected cells allow a sustained viral replication that leads to the release of virus into the bloodstream that can be in excess of 10^12^ genome copies/mL, and undergo apoptosis, with the consequence of a temporary block in erythropoiesis that can be clinically relevant [3,4]. The viraemic phase is followed by systemic distribution of the virus to other non-erythroid cell types, including endothelial, stromal, or synovial cells, that are also susceptible but mainly non-permissive [5]. In these cells, infection can trigger inflammatory responses and consequent tissue damage, leading for example to the common clinical presentations of erythema infectiosum and arthritis/arthralgia, and generally resulting in long-term persistence of viral DNA within tissues [6]. The development of a neutralising immune response is functional to the clearance of the virus from the blood and termination of infection, but may also contribute to pathogenesis in peripheral tissues [7].

The genome of B19V is a ssDNA molecule, of either polarity, 5596 nt long, composed of two terminal regions, 383 nt, that provide the origins of replication, flanking a unique internal region, 4830 nt, containing all open reading frames. Three genotypes (1–3) have been recognised for B19V [8,9]. Genotype 1 is prevalent worldwide [10], apparently having replaced, in the last fifty years, genotype 2 [11], which is now found sporadically [12], while genotype 3 can be found at lower frequency in restricted geographic areas [10]. Genetic distance between the three different genotypes is in the order of 10%, while intragenotype distance values are different—higher for genotypes 2 and 3 (3–8%), lower for genotype 1 (1–3%)—possibly reflecting a shorter evolutionary history for the latter [9,13]. As of present, research did not reveal differences in the biological [14], immunological or pathogenetic characteristics among the different genotypes [15], except for the closer association of genotype 2 with tissue persistence in elder people [11,16]. Experimentally, sequence determination of B19V DNA from clinical samples is frequently reported in literature to assign genotype and for finer molecular epidemiological tracing. Quite predictably, isolate clusters can be distinguished mainly based on sample population composition, but due to limited investigation, a comprehensive picture of genetic diversity within B19V is still lacking. Based on the available data, a relatively high mutation/substitution rate has been predicted for B19V, in the order of 10^−4^ and similar to other ssDNA viruses, implying high intrinsic genetic diversity and evolutionary potential [17,18]. However, such prediction is in some contrast to experimental data showing a more conserved evolutionary pathway over longer time periods [19,20]. Moreover, high rates would also predict a high heterogeneity and dynamicity of viral populations as a result of the replicative process, therefore implying a quasispecies structure [21], a hypothesis that can be tested.

Technically, until now, genome sequencing has been mainly carried out by a standard Sanger sequencing technique, though the increasing availability and use of Next Generation Sequencing (NGS) with its depth of coverage incorporates additional relevant information on intrinsic sequence heterogeneity [22,23]. However, integration of this information in analysis of sequence variation is not routinely obtained. The present work has been carried out as a first exploratory study to investigate genomic sequence heterogeneity within and between B19V isolates, by application of NGS techniques, and by the development of a novel dedicated bioinformatic tool and analysis pipeline. As a result, experimental output yielded the following: (i) for each isolate, representation of NGS sequence data via a position-specific probability matrix, and an assessment of genomic heterogeneity using Shannon entropy as an index of intra-sample diversity; (ii) for the set of isolates, evaluation of diversity among the position-specific probability matrices, and an assessment of genetic distances by indexes combining both intra- and inter-sample diversity. This information can yield a finer insight into the genetics of B19V, and in perspective can be incorporated into a theoretical approach conforming to quasispecies theory.

## 2. Materials and Methods

**Samples.** Reference samples, deriving from the consensus B19V EC genotype 1 sequence (GenBank KY940273.1) and the related synthetic genetic system previously developed [24], included: (i) cloned DNA, excised from plasmid CJ0, as an in-process control; ii) a B19V laboratory strain stock sample, EC1622, propagated in vitro in differentiated erythroid progenitor cells as described. A panel of high-titre B19V viraemic serum samples (>10^6^ genome copies/mL) was collected in the course of institutional diagnostic service at the Microbiology Unit, S. Orsola Hospital, Bologna, in the period 2012–2020. Samples were available for virologic investigation according to institutional guidelines and compliance with Italian Privacy law, for the sole purpose of viral DNA sequencing, waiving patient informed consent.

**Sample processing.** For each sample, a 100 µL volume was processed by Maxwell Viral Total Nucleic Acid Kit (Promega) on a Maxwell MDX platform, to obtain a purified total nucleic acid fraction. For sequencing, a target genomic region spanning positions 2210–3342 was selected, corresponding to the region between A1.2 splice acceptor and pAp2 cleavage-polyadenylation signals. Target DNA was amplified by high-fidelity PCR, using primers R2210 (forward) and R3342 (reverse) [25,26]. Amplification was carried out by using the High Fidelity PCR System (Roche), according to manufacturer’s instructions, with a thermal profile consisting of initial denaturation 94 °C, 2′; 10 cycles of denaturation 94 °C, 15″, annealing 50 °C, 30″, extension 72 °C, 2′30″; 20 cycles of denaturation 94 °C, 15″, annealing 50 °C, 30″, extension 72 °C, 2′30″ incremented by 5″/cycle; final extension 72 °C, 7′. Following agarose gel electrophoresis analysis, the amplification product was purified from the reaction volume by using a Wizard^®^ SV Gel and PCR Clean-Up System kit (Promega), and finally quantified by the Qubit 2.0 Fluorometer (Promega). For each sample, a minimal amount of 0.2 µg DNA was processed for sequencing.

**Next Generation Sequencing.** Library preparations and NGS were carried out by an external service (IGA Technology, Udine, Italy). Following DNA fragmentation, library preparation was made with Celero DNA-Seq kit (Tecan). Both input and final libraries were quantified by Qubit 2.0 Fluorometer, and quality tested by the Agilent 2100 Bioanalyzer Sensitivity DNA assay. Libraries were then sequenced on Illumina NovaSeq 6000 in paired-end mode, with reads of 120 bps. The preliminary analyses performed were base calling and demultiplexing, by the Bcl2Fasq 2.20 version of the Illumina pipeline, followed by adapters masking using Cutadapt v1.11 from raw fastq data. As an output, IGA returned fastq reads files for further data processing conducted in our laboratory.

**Sequence Data Processing.** Received fastq reads files were examined for Quality control by FastQC; then, high-quality reads were processed by trimming (Trim Galore!), whereas duplicate removal (Prinseq v0.20.4) was not necessary. Alignment of high-quality reads and genome indexing was performed using BowTie2 v2.5.0. The reference sequence used for these operations is the consensus B19V EC, genotype 1, previously developed by the research group (GenBank KY940273.1). After alignment, the SAM files generated by BowTie2 were converted into BAM files via SAMTools, to allow visualisation of the alignment on Integrative Genomics Viewer (IGV) [27]. For a preliminary analysis on genome variability, individual consensus sequences were obtained starting from the multiple alignment files by a dedicated pipeline, using different functions from SAMtools v1.16.1, BCFtools v1.15, and setqk v1.2-r94. The resulting fasta files with individual consensus sequences could be imported into MEGA11 software [28] for alignment and further analysis.

**Sequence Data Analysis.** NGS data were analysed by an in-house developed tool, QSA (Quasi-Species Analyser). QSA, still in its beta version (https://github.com/ovaltriangle/qsa, accessed on 2 January 2023), was specifically developed within this project to carry out analyses on sequence variability data embedded in BAM files. QSA is a unique tool, whose functions allow: (i) processing of aligned reads from BAM files to create a position frequency and a position probability matrix; (ii) calculation of position-specific normalised Shannon entropy values (called α-diversity) and obtainment of a graphical display; (iii) calculation of aggregate, inter-sample entropy values (called ‘δ-diversity); (iv) calculation of inter-sample genetic distance based on inter-sample entropy values (called ‘σ-diversity’). Further analysis and graphical elaboration were carried out using Python on IDLE Spyder (packages numpy, pandas, matplotlib for boxplot analysis) [29] and R Studio (packages stringr, pvclust for hierarchical clustering, cluster for K-means analysis) (https://posit.co/, accessed on 2 January 2023).

**Data availability.** NGS raw fastq reads have been submitted to the European Nucleotide Archive, Study ID PRJEB58863 (ERP143941).

## 3. Results

### 3.1. Samples and NGS Output

**Figure 1:**
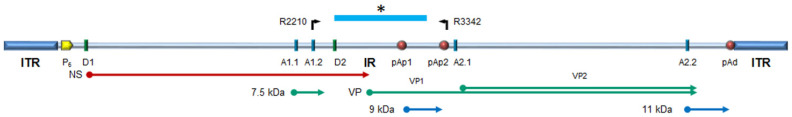
Schematic diagram of B19V genome. ITR, inverted terminal regions; IR, internal region; cis-acting functional sites: P6, promoter; pAp1, pAp2, proximal cleavage-polyadenylation sites; pAd, distal cleavage-polyadenylation site; D1, D2, splice donor sites; A1.1, A1.2, A2.1, A2.2, splice acceptor sites. Top: primer location and genome segment included in NGS analysis (*). Bottom: open reading frames and coding sequences for the viral proteins. NS, non-structural protein NS1; VP, structural proteins, colinear VP1 and VP2, assembled in a T1 icosahedral capsid; 7.5 kDa, 9.0 kDa, 11 kDa: minor non-structural proteins. Modified from [30].

A total of 26 samples were included in the study (Table 1). Reference samples, deriving from the consensus B19V EC genotype 1 sequence and the related synthetic genetic system previously developed, included: (i) cloned DNA, excised from plasmid CJ0, as an in-process control; (ii) a B19V laboratory strain stock sample, EC1622, propagated in vitro in differentiated erythroid progenitor cells as described. Tested clinical samples consisted of a panel of 24 high-titre B19V viraemic serum samples (>10^6^ genome copies/mL), collected in the course of an institutional diagnostic service at the Microbiology Unit, S.Orsola Hospital, Bologna, in the period 2012–2020. For investigation, a target genomic region spanning positions 2210–3342 was selected, corresponding to the region between A1.2 splice acceptor and pAp2 cleavage-polyadenylation signals, encompassing the VP1 N-terminal unique region (Figure 1). Target was amplified by high-fidelity PCR, the resulting amplification products were purified and then processed for NGS, first by DNA fragmentation and library preparation, then by sequencing on Illumina NovaSeq 6000 in paired-end mode, with reads of 120 bps length. As output for each sample, reads received were in the range 0.82–6.0 × 10^6^, yielding a depth of coverage in the range 0.87–6.3 × 10^5^ counts per position. Quality control on reads reported a high overall score, excluding systematic sequencing errors, so all of the obtained reads were used for further analysis.

### 3.2. Sequence Alignment

Reads obtained from each individual sample were aligned to the B19V EC genotype 1 reference sequence. B19V EC is a genotype 1 consensus, obtained from alignment of a set of 50 genomic-length sequences, of representative isolates collected in different areas ante year 2010, and coincident with a possible ancestral state as determined from an ML phylogenetic tree [24]. Alignment generated individual consensus sequences that were imported into MEGA11 software for visualisation, further alignment, and investigation of the presence of sequence variants with respect to the reference sequence, calculation of pairwise genetic distances and graphical representation of distances. For the purpose of our work, results provided a comparison term to further analysis aimed at incorporating depth of sequencing as obtained from NGS.

At first, analysis of NGS-derived consensus sequences confirmed the perfect identity of the reference CJ0 and EC1622 samples to the B19V EC genotype 1 reference sequence from which they were derived. Then, all of the clinical samples, except sample S20, could be aligned to this same genotype 1 reference sequence. For each genotype 1 sample, a range of 2–14 individual base differences were found, dispersed over 47 base positions, 6 of which were common to more than 12 samples. Sample S20 was a notable exception, showing 53 base differences to consensus, with only 9 being in common with other samples, and unexpectedly turning out to align with a genotype 2 consensus sequence (Table 1) (Supplemental Table S1).

### 3.3. Sequence Variability Analysis

In the analysis of the individual consensus sequences only, all information on sequence diversity embedded in reads obtained from NGS techniques is lost. IGV allows for visual inspection of diversity at individual positions for each obtained read, but cannot allow for further aggregate analysis. For the purpose of incorporating NGS information in the analysis of intra-sample sequence variability, a novel algorithm was carried out by using a specific, in-house developed bioinformatic tool, QSA, and a related analysis pipeline.

For the scope of this work, to ensure homogenous coverage and avoid background noise linked to reads misalignment, an effect observed at the extremes of the sequenced products, all samples were analysed to a restricted sequence—spanning position 2390 to 3242, for a total of 852 base positions. For each single sample, aligned reads in BAM files were processed by QSA to create first a position frequency matrix (PFM), and then a position probability matrix (PPM). PPM gives information on the normalised probability of occupation at each position by each one of the bases. On the PPM matrix, for each position i, and $j\in \left\{T,C,G,A\right\}$, $\left[j\right]=4,$ a normalised Shannon entropy was calculated as:

$$\eta (i)=\frac{-\sum_{j}p_{ij}\log_{2}p_{ij}}{\log_{2}\left[j\right]}$$

**Figure 2:**
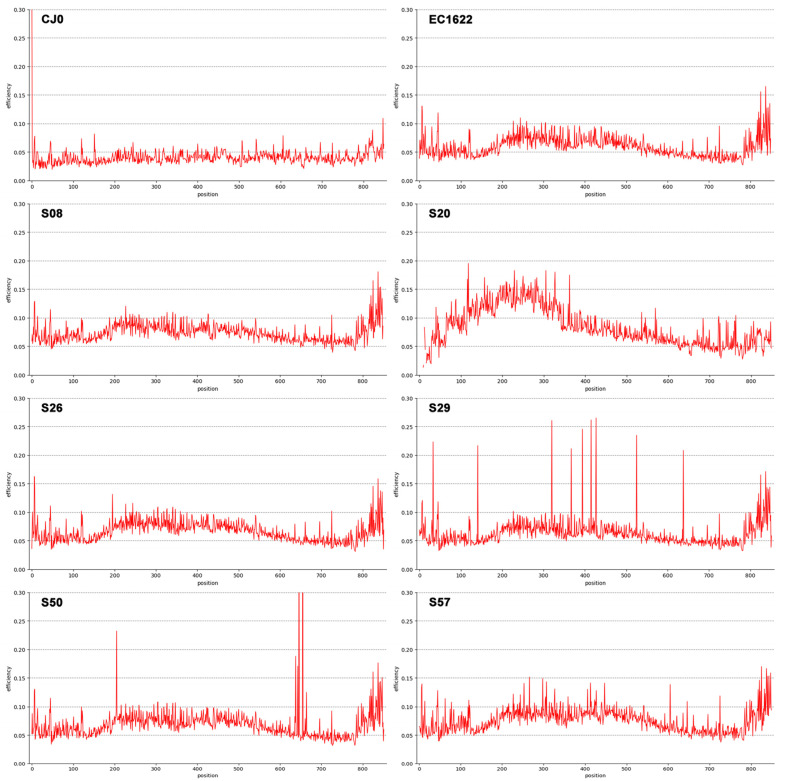
Line graph representation of Shannon Entropy Efficiency values in the genome segment from nt. 2390 to nt. 3242. A selection of representative samples is shown (see also Figure 3 for the complete set). CJ0 is the reference, control plasmid DNA. EC1622 is a reference B19V laboratory strain stock sample. S08, S20, S26, S29, S50 and S57 are clinical isolates. Low efficiency values and low dispersion are typical for control CJ0, virus stock EC1622 and also evident in samples S08 and S26. Higher efficiency and dispersed values can be observed for samples S20, S29, S50 and S57. In particular, sample S20 is a genotype 2 isolate showing higher variability in the first 400 nts; samples S29 and S50 show relatively low efficiency values, but distinct position-specific peaks that can be interpreted as polymorphisms; sample S57 shows a relatively higher efficiency value along with higher, more distributed dispersion.

**Figure 3:**
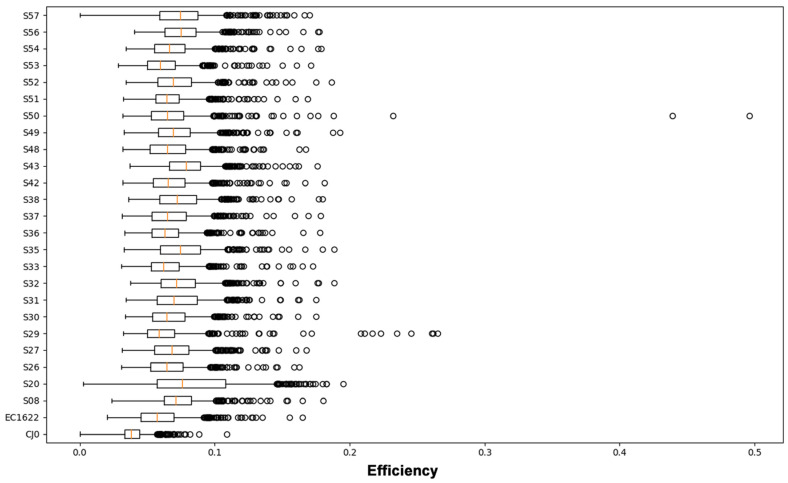
Boxplot representation of the distribution of Shannon Entropy Efficiency values in the genome segment from nt. 2390 to nt. 3242, each position individually contributing. For each sample, the average, 25–75% interquartile range, SD interval and single high-range outlier values are shown. Average value is termed as ‘α-diversity’ index. For interpretation of data, see also Figure 2.

The quantity $\eta_{i}$, termed as efficiency, is a normalisation of Shannon’s entropy to assess position-specific variability in a set of aligned sequences, in this case obtained from the totality of NGS reads. First, Shannon’s entropy values are calculated at each position in the sequence. Afterwards, values are divided by the maximum value of Shannon’s entropy (in binary notation, 2 bits) to find the efficiency at each position. Efficiency values at each position can be represented as a line graph over the sequence length (Figure 2), while values distribution can be calculated and represented in a box-plot graph (Figure 3).

The overall sum of the entropy values at each position in the sequence (total efficiency), normalised by the sequence’s length, yields an averaged quantity that can be named an ‘α-diversity’ index (Table 1). Considering the reference samples, CJ0 is a plasmid-derived insert, while EC1622 is representative of an actual replicating viral population, where some variation is expected to occur. Thus, α-diversity of CJ0 was at the lowest, while the higher value in EC1622 likely reflects this fact. Only for the genotype 1 samples, the mean α-diversity was 0.070 (range 0.062–0.079); the genotype 2 sample showed indeed the highest value of 0.083 (Table 1). The α-diversity index is a unique value related to overall genomic variability, while the distribution of efficiency values yields indication on sequence homogeneity. Low or high outlier values indicate more conserved or variable positions on the sequence that can be identified on the line graph representation. It should be remarked how these quantities only refer to position specific variability as detectable from the totality of aligned NGS reads, not taking into consideration any possible linkage into whole-length genomic sequences as a means of reconstruction of individual sequences’ identity and abundance.

### 3.4. Sequence Distances Analysis

**Figure 4:**
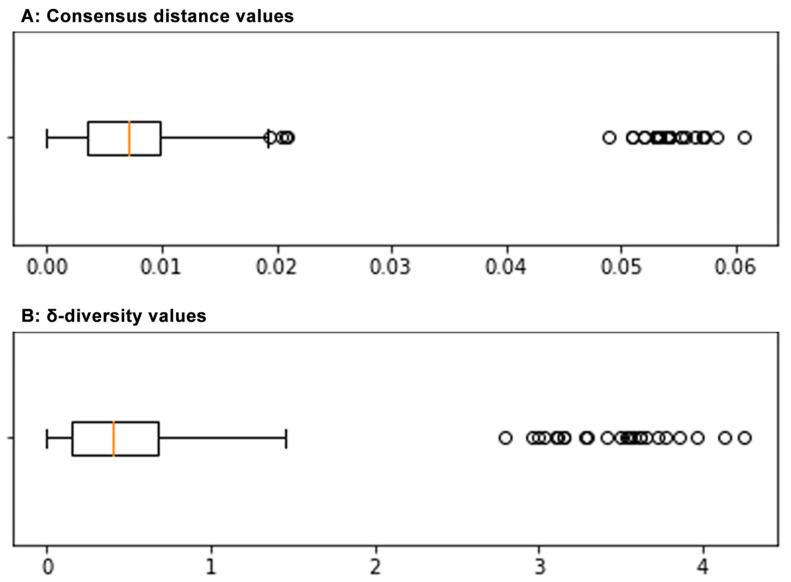
Boxplot graph of the distribution of consensus distance values (**A**) and of δ-diversity values (**B**) in the sample set. For both, average, 25–75% interquartile range, SD interval and single high-range outlier values are shown. Average δ-diversity value is termed as ‘σ-diversity’ index.

For the purpose of inter-sample diversity analysis, NGS-derived consensus sequences were first analysed by using the functions available in MEGA11 software. A pairwise distance plot was constructed under an MCL (Maximum Composite Likelihood, gamma-distributed) substitution model. The mean normalised distance of genotype 1 samples to CJ0/EC1622 reference sequences was 0.007 (range 0.005–0.012), similar to the mean of normalised distances within samples that was 0.007 (range 0.004–0.011). The genotype 2 sample was an evident outlier with a distance to reference sequence of 0.051, and a mean distance to genotype 1 samples of 0.055 (range 0.052–0.058) (Table 1) (Figure 4) (Supplemental Table S2).

For the same purpose of inter-sample diversity analysis, to exploit the information embedded in reads obtained from NGS techniques, a measure analogous to a genetic distance was calculated on the basis of the position probability matrices previously obtained, thus incorporating both intra- and inter-sequence variability. Comparison between any two samples was carried out by calculating the Manhattan Distance, that is, the difference matrix between each of the samples’ PPMs, as a measure of sequence diversity over each single position. Then, for each difference matrix, a total normalised efficiency value was obtained as a measure of pairwise genetic distance, a quantity that could be termed ‘δ-diversity’. Repeating the procedure for all pairs, a cumulative distance matrix was obtained, analogous to a standard genetic distance matrix, but critically incorporating all position-specific information as derived from the NGS output. Such a distance matrix, implying both intra- and inter-sample diversity, yields information on what can be named ‘σ-diversity’ in a set of sequences. The σ-diversity in a set of sequences can be calculated as the normalised amount of δ-diversity, or diversity between α-diversity values.

The mean δ-diversity values, normalised to 100 nt positions, of genotype 1 samples to CJ0/EC1622 reference sequences were 0.94–1.06, higher than the mean of normalised distances within samples that was 0.51 (range 0.38–0.87). The genotype 2 sample had a mean diversity to reference sequences of 4.08, and a mean diversity to genotype 1 samples of 3.09 (range 2.22–3.89) (Table 1) (Figure 4) (Supplemental Table S2).

**Figure 5:**
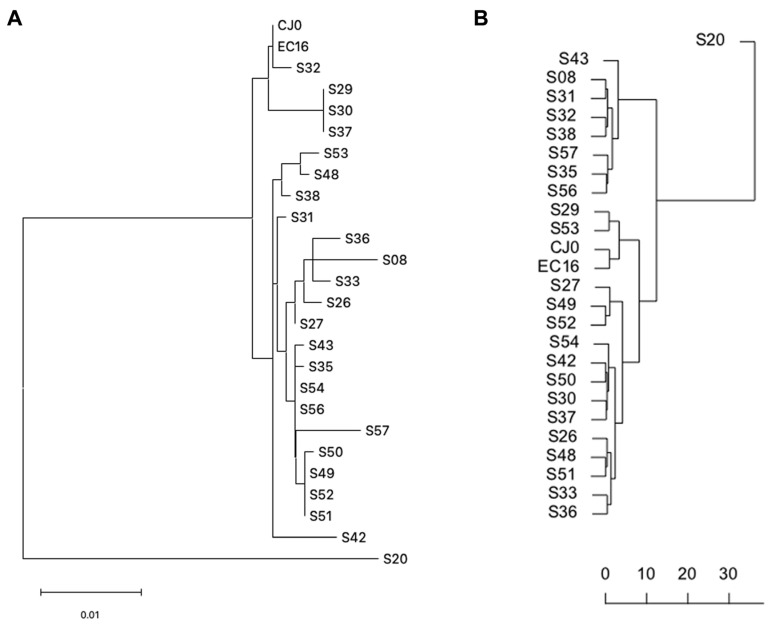
Dendrogram representation of consensus distances and δ-diversity values, hierarchical clustering. (**A**) Distance based dendrogram (MEGA 11); (**B**) δ-diversity based dendrogram (PVClust).

A dendrogram representation of distances was built based on both the consensus distance and δ-diversity matrices of values, by Hierarchical Clustering (Figure 5A,B). Since the source matrices are different, the resulting tree topology is not equivalent. However, in the case of the distance based dendrogram (5A), the resulting topology is not supported by bootstrap analysis, while the δ-diversity based dendrogram (5B) has supporting unbiased bootstrap *p* values for nodes > 0.96.

**Figure 6:**
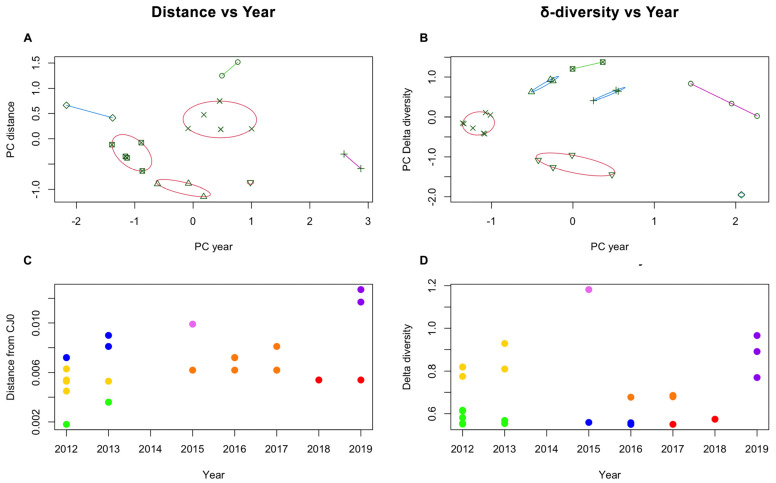
K-means analysis of sample clustering, based on either consensus distances (**A**,**C**) or δ-diversity values (**B**,**D**). Principal Component (PC) analysis indicates clustering according to year of isolation, and best separation was achieved when K = 7 for both datasets. Groups are separated on the PC graphs (**A**,**B**), and identified by colour code in the year vs. distance graphs (**C**,**D**). While the distribution of each isolate among groups is not equivalent for the two datasets, a similar dependence on year of isolation suggests a temporal replacement pattern of different isolate groups.

To further investigate any possible correlation within the sample set, K-means analysis was carried out on both datasets. While average distance and δ-diversity did not yield separate clusters, discrete clustering was obtained when considering the year of isolation and either average distance or δ-diversity. When analysing genotype 1 samples only, the best separation was achieved by partitioning the sample set in seven subsets (Figure 6A,B). Given the lack of direct correlation between the two variables, the distribution of subgroups was not identical for the two datasets, but in both cases, analysis suggests the occurrence of a series of epidemiologically related isolate clusters in a distinct temporal replacement pattern (Figure 6C,D).

## 4. Discussion

NGS techniques are increasingly replacing Sanger sequencing techniques in virologic applications: from sequencing of individual isolates to detection of variants of interest; from diagnostics in clinical settings to virus discovery and comprehensive virome analysis; and last but not least, for wide epidemiological surveillance and preparedness for novel pandemic threats [31]. The enormously increased amount of information obtained from NGS, compared to Sanger sequencing, provides an opportunity for expanded knowledge and insights into system biology and reconstruction of virus evolution.

In addition to the opportunity of whole genome sequencing and identification, high-coverage NGS can provide information on the amount of sequence heterogeneity that can be found within a viral population, one of the key parameters directing viral adaption and evolution, including evolving relationships with hosts [32]. Although NGS can be carried out directly on clinical samples in a ‘metagenomic’ approach, this may not be suitable to detect sequence heterogeneity within a viral population representing a low-fraction of the accessible targets. For this purpose, a higher depth of coverage is needed; thus an amplification step of the target of interest is required, with the necessary assumption that the original sequence diversity is represented in the amplification product [31].

Although the high output content is a key asset of NGS techniques, information on sequence heterogeneity is not easily incorporated into downstream analysis. In most cases, NGS reads are aligned against a reference sequence and a novel consensus is obtained, which can highlight majority variants with respect to the reference. However, the presence and distribution of minority variants, whose information is embedded into NGS output, is not easily extrapolated and available as information for deeper analysis [33]. Further, several bioinformatic tools are available that can attempt at reconstructing viral genomes by progressive assembly of reads; in this case, the output is a predicted partition of the total set of different viral sequences into subsets of more homogenous sequences [34]. However, short reads, as obtained from Illumina sequencing, cannot be definitely assigned to the same template on the basis of variation patterns; on the other hand, longer reads, as can be obtained for example by Nanopore techniques, often have a higher error rate, being thus unsuitable for the analysis of minority variants. Given these limits, a compromise in the informative potential of NGS must be sought [35].

In the frame of the quasispecies theory, a viral population needs to be considered as a set of variant sequences [21]. The complexity of a viral population is determined by the number and relative abundance of the different subsets of variant sequences, as well by the degree of sequence diversity among variants [36]. In a quantitative approach, complexity indexes usually refer to quasispecies composition, given the number and relative frequency of variant sequences. This approach requires a reliable reconstruction of variant genomes, which by themselves are not experimentally determined data. On the other hand, converting an alignment of reads into a position-specific frequency matrix, an index of sequence complexity can also be obtained by evaluating the degree of heterogeneity at single positions within the viral population; such analysis would bring the advantage of being accurately descriptive and not relying on inferential algorithms, usually with a low predictive value when compared. A generally accepted index such as Shannon entropy can be successfully adapted and exploited for this purpose.

Given such considerations, and in the pursuit of a more informative approach exploiting NGS data, we developed a novel bioinformatic tool, named QSA. It is unique in its functions, and has established a dedicated analysis pipeline for investigation of viral sequence heterogeneity. The challenge was in the exploration of sequence diversity embedded within NGS reads, as compared to information obtained from standard, consensus-based analysis. Starting from aligned NGS reads, via the construction of position-specific probability matrixes, the experimental pipeline yielded information on two inherent basic parameters. The first, which we defined as α-diversity, is a measure of intra-sample sequence heterogeneity, derived from the amount and distribution of position-specific, normalised Shannon Entropy. The second, which we defined as σ-diversity, is a combined measure of intra- and inter-sample sequence heterogeneity, derived from the amount of inter-sample sequence heterogeneity, defined as δ-diversity, also incorporating the amount and distribution of samples’ α-diversity. While α-diversity is a parameter that characterises each viral population and can be considered in addition to the definition of a unique, individual consensus sequence, the δ-diversity and σ-diversity parameters incorporate both intra- and inter-sample diversity, and can be considered in comparison to other measures of genetic distances, normally derived from unique consensus sequences. In this way, information from NGS data becomes incorporated into genomic information.

Concerning the specific aim of investigating sequence heterogeneity within B19V, by using this conceptual approach and bioinformatic tools, results yielded the following information: (i) the reference strain stock yielded the expected consensus sequence, without any mutation; its α-diversity was in the lowest range of observed experimental values, 0.060, thus confirming stability of the genome in the experimental system; (ii) clinical isolates, genotype 1, also showed low α-diversity values, in the range 0.062–0.079, while a few samples showed distinct position-specific polymorphisms; polymorphisms at specific positions are suggestive of the emergence of minority variants, although the presence of a coinfecting cell population cannot be formally excluded by NGS analysis; (iii) distance values of genotype 1 samples within the sample set and with respect to reference samples are comparable, thus confirming the characteristics of the consensus-derived reference strain; however, diversity values are lower within the sample set than to reference samples, thus suggesting different evolutionary dynamics; (iv) a few genetically related clusters emerged when analysing inter-sample distances, correlated to the year of isolation; (v) the single genotype 2 isolate showed the highest efficiency value, 0.083, even if not presenting polymorphisms, and was an evident outlier when analysing inter-sample distance and diversity.

Implications of the results are that the accumulation of diversity in B19V occurs at a low pace. Intra-sample diversity is low; the data can be anticipated because of the dependence of the virus on cellular DNA polymerase for its replication. Inter-sample diversity is also low considering the distance between the reference samples, which are based on a consensus, possibly ‘ancestral’ sequence, and the clinical isolates collected in the past ten years over a defined geographical setting. Higher substitution rates, as reported in the literature, are probably an overestimate, possibly due to a confounding effect of temporal and spatial heterogeneity in sampling [37]. However, statistical analysis suggests that more closely related isolate clusters can circulate in defined temporal patterns, an observation favoured by the close geographical area of sample isolation. Interestingly, the genotype 2 isolate has a higher intrinsic heterogeneity, a phenomenon possibly linked to an association in the host, in a long-term persistent/latent state, followed by reactivation and high-titre viraemia as detected in the collected sample (since a de novo, exogenous infection can be deemed most unlikely in our case, although not formally excluded). Extension of the investigation to the whole genome, rather than to a limited genome segment, and inclusion of more numerous and diverse samples is required in future research to validate the model, extend validity of α- and σ-diversity parameters and derive a clearer picture of B19V sequence diversity and evolution.

In conclusion, NGS analysis is an effective tool for investigating sequence diversity, itself an observable that can be incorporated into the quasispecies theory framework to yield a better insight into viral evolution dynamics. The bioinformatic tool and pipeline that we developed and used in the present work can be considered functional to this aim.

## Figures and Tables

**Table 1:** Sample set in the study and derived distance and diversity values.

Sample ^1^	Date	Viral Load	Variations ^2^	Distance to CJ0 ^3^	Mean Distance ^3,5^	α-Diversity ^4^	δ-Diversity ^4,5^
CJ0	---	1.00 × 10^6^	0	0.0000	0.0067	0.0403	0.9376
EC1622	---	1.00 × 10^6^	0	0.0000	0.0067	0.0595	1.0601
S08	04/09/2019	8.26 × 10^6^	14	0.0127	0.0105	0.0735	0.4695
S26	21/03/2012	1.00 × 10^10^	8	0.0072	0.0063	0.0663	0.4555
S27	14/04/2012	1.20 × 10^8^	6	0.0054	0.0047	0.0699	0.3891
S29	11/06/2012	5.40 × 10^6^	6	0.0053	0.0099	0.0635	0.6682
S30	14/06/2012	5.00 × 10^9^	6	0.0053	0.0099	0.0674	0.4018
S31	15/06/2012	6.00 × 10^9^	5	0.0045	0.0052	0.0737	0.4735
S32	27/06/2012	2.00 × 10^6^	2	0.0018	0.0084	0.0744	0.5090
S33	06/11/2012	2.00 × 10^10^	10	0.0063	0.0050	0.0647	0.5663
S35	06/06/2013	1.00 × 10^10^	7	0.0081	0.0070	0.0759	0.6108
S36	25/06/2013	1.00 × 10^10^	11	0.0090	0.0079	0.0654	0.5164
S37	12/09/2013	1.00 × 10^9^	6	0.0053	0.0099	0.0677	0.3932
S38	26/09/2013	1.00 × 10^7^	4	0.0036	0.0056	0.0742	0.5011
S42	07/05/2015	3.00 × 10^6^	11	0.0099	0.0098	0.0681	0.3863
S43	27/06/2015	1.00 × 10^6^	7	0.0062	0.0050	0.0787	0.8703
S48	27/01/2016	1.50 × 10^7^	8	0.0072	0.0074	0.0670	0.4206
S49	03/03/2016	2.00 × 10^6^	7	0.0062	0.0046	0.0716	0.4116
S50	23/08/2016	3.00 × 10^6^	7	0.0072	0.0054	0.0680	0.3872
S51	23/03/2017	1.00 × 10^10^	7	0.0062	0.0046	0.0669	0.4247
S52	01/06/2017	2.00 × 10^8^	7	0.0062	0.0046	0.0717	0.4125
S53	29/07/2017	5.00 × 10^7^	8	0.0081	0.0081	0.0620	0.8029
S54	28/11/2018	1.86 × 10^6^	6	0.0054	0.0042	0.0687	0.3837
S56	26/06/2019	2.69 × 10^6^	6	0.0054	0.0042	0.0763	0.6484
S57	23/07/2019	2.96 × 10^6^	13	0.0117	0.0100	0.0754	0.5745
Mean for Genotype 1			7	0.0067	0.0069	0.0700	0.5077
S20	02/05/2020	7.65 × 10^6^	53	0.0509	0.0547	0.0828	3.0858

^1^ CJ0 is the reference plasmid DNA, EC1622 is a reference virus stock. All samples (S08–S57) are genotype 1 with the exception of sample S20, which is genotype 2. ^2^ For all samples, variations are nucleotide differences with respect to the reference sequence (KY940273.1). ^3^ Distance to CJ0 and mean distance calculated under MCL substitution model by using MEGA 11 software. ^4^ α-diversity and δ-diversity values calculated as described in the text. ^5^ Mean distance and δ-diversity values for CJ0, EC1622 and S20 are calculated with respect to genotype 1 samples; genotype 1 sample values are calculated excluding CJ0, EC1622. and S20.

## Data Availability

NGS raw fastq reads have been submitted to the European Nucleotide Archive, Study ID PRJEB58863 (ERP143941).

## Supplementary Materials

The following supporting information can be downloaded at: https://www.mdpi.com/article/10.3390/v15010217/s1, Table S1: consensus sequence nt and aa variations; Table S2: MEGA11 genetic distance and NGS δ-diversity values matrices.
